# Tolerogenic Immunoregulation towards Salmonella Enteritidis Contributes to Colonization Persistence in Young Chicks

**DOI:** 10.1128/IAI.00736-20

**Published:** 2021-07-15

**Authors:** Khin K. Z. Mon, Colin Kern, Ganrea Chanthavixay, Ying Wang, Huaijun Zhou

**Affiliations:** a Department of Animal Science, University of California, Davis, California, USA; University of California San Diego School of Medicine

**Keywords:** RNA-seq, *Salmonella*, transcriptome, persistent infection, immune response, chicken

## Abstract

Long-term survival and the persistence of bacteria in the host suggest either host unresponsiveness or induction of an immunological tolerant response to the pathogen. The role of the host immunological response to persistent colonization of Salmonella Enteritidis (SE) in chickens remains poorly understood. In the current study, we performed a cecal tonsil transcriptome analysis in a model of SE persistent infection in 2-week-old chickens to comprehensively examine the dynamics of host immunological responses in the chicken gastrointestinal tract. Our results revealed overall host tolerogenic adaptive immune regulation in a major gut-associated lymphoid tissue, the cecal tonsil, during SE infection. Specifically, we observed consistent downregulation of the metallothionein 4 gene at all four postinfection time points (3, 7, 14, and 21 days postinfection [dpi]), which suggested potential pathogen-associated manipulation of the host zinc regulation as well as a possible immune modulatory effect. Furthermore, delayed activation in the B cell receptor signaling pathway and failure to sustain its active state during the lag phase of infection were further supported by an insignificant production of both intestinal and circulatory antibodies. Tug-of-war for interleukin 2 (IL-2) regulation between effector T cells and regulatory T cells appears to have consequences for upregulation in the transducer of ERBB2 (TOB) pathway, a negative regulator of T cell proliferation. In conclusion, this work highlights the overall host tolerogenic immune response that promotes persistent colonization by SE in young layer chicks.

## INTRODUCTION

Salmonella enterica serovar-associated infection in chicken host acts as the main source of foodborne illness in the human population, not only leading to significant economic losses in the poultry industry but also causing major health concerns for consumers of poultry products. The Foodborne Diseases Active Surveillance Network reported in 2013 that 20% of the reported 9.4 million cases of foodborne illness were caused by Salmonella-contaminated food sources, which accounted for 26% of the hospitalized cases (1). In the chicken host, acute/fatal and chronic salmonellosis are the two most common types of illness, depending on the age and immune competence of the host and the infecting Salmonella serovars (2, 3). Chronic salmonellosis is a typical subclinical illness observed in week-old or older chicks infected with Salmonella enterica subsp. *enterica* serovar Enteritidis (SE) in which the host does not display any obvious clinical symptoms or discomfort (4). Instead, prolonged colonization by SE of the gastrointestinal tract leads to fecal shedding of the bacterial organism in the environment, which is a classic characteristic of chronic salmonellosis in the chicken host (5). SE fecal excretion from the infected host into the environment then acts as a contaminant source for further transmission of Salmonella to naive hosts. Prolonged persistence of SE within the gastrointestinal tract of the infected host suggests pathogen-driven adjustment and modulation of the host immune response to accommodate the long-term survival of the organism.

Gut-associated lymphoid tissue (GALT) is the important immunological system in the gastrointestinal tract of the chicken host that plays an important role in inducing an appropriate immunological response against pathogens. As part of the GALT, the cecal tonsil is a lymphoid tissue that progressively develops into an immunologically mature organ after the first few weeks of a chick’s life. Initially, at hatching, the gastrointestinal tract of the avian host is not yet fully developed, both anatomically and functionally (6). The lack of a competent adaptive immune system in newly hatched chicks also results in their being highly susceptible to infection (7), often leading to a high mortality rate. Partial protection conferred by the existing innate immune system as well as passive immunity transferred from the maternal parent is often not sufficient to fight off infection. However, after the first week of life, chicks undergo dramatic changes in both intestinal and adaptive immune function development in parallel with gut colonization by commensal microorganisms (8). Development of GALT, maturation of acquired intestinal immune functions, and establishment of the gut microbiome are key host processes required for maintaining homeostasis while providing colonization resistance against pathogenic organisms. At hatching, GALT contains functionally immature T and B cells. The rapid development of immune competence and functionality of immune cells in avian GALT occurs over the first 2 weeks of a chick’s life (7). Exposures to environmental antigens as well as feed intake in the posthatching period are presumed to be the main stimulants in triggering the initial activation of the immune maturation process. Therefore, the first 2 weeks of a chick’s life is the critical period for the avian host to develop an adequate immune system to defend against invasion by pathogenic organisms. Analysis of exposure to an enteric pathogen like SE following this critical 2 weeks of the posthatching period will provide novel insight into the regulation and mechanism of an adequate adaptive immune response to intestinal pathogen colonization in chickens.

Although there is no clear clinical sign associated with SE infection in the chick host, alteration in host immune-related gene expression following the infection suggests its pathogenic characteristic ability to induce the host response. Activation of the host innate immune response was detected immediately following Salmonella infection in a day-old chick, with increased production of proinflammatory cytokines and in chemokines that lead to migration of heterophils and macrophages to infection sites and a marked increase in host inflammatory response (9–12). Concerning the adaptive immune response, Salmonella infection in a chicken host was reported to elicit both antibody response and cell-mediated immune response (13). However, the contribution of both arms of adaptive response to pathogen clearance or reduction in colonization in the gastrointestinal tract of the host during primary infection remains unclear. The absence of effective adaptive immune responsiveness may contribute in part to persistent colonization by SE of the gut of the chick host. Therefore, we hypothesized that tolerogenic response of host adaptive immune function is a major contributing factor in colonization persistence of SE infection in the gastrointestinal tract of 2-week-old layer chicks.

## RESULTS

### SE colonization persistence phenotype in the cecum of 2-week-old layer chicks.

**FIG 1:**
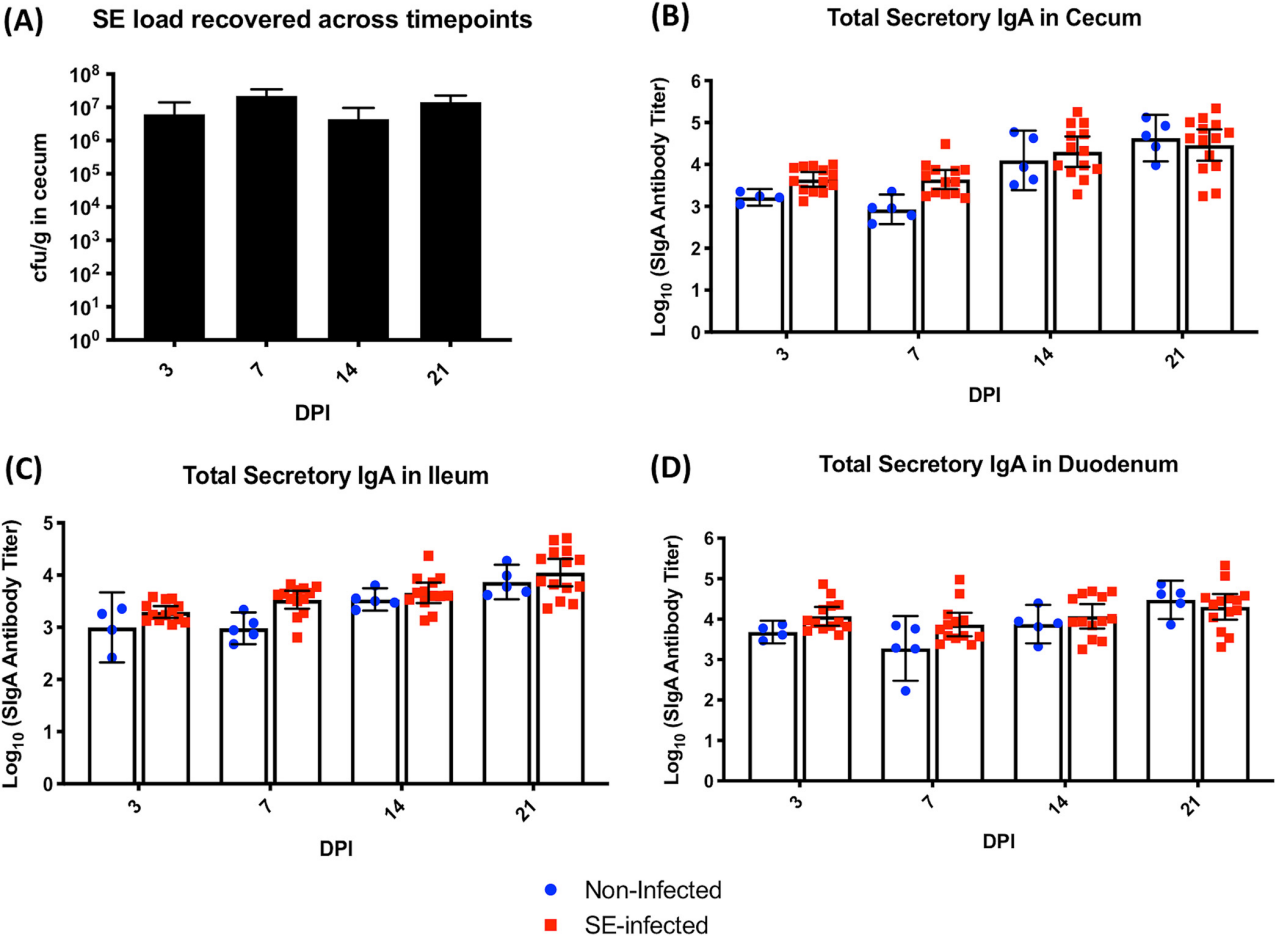
(A) Persistent high bacterial load of SE across four postinfection time points in the cecum. Geometric means of bacterial loads recovered from cecum (CFU per gram of tissue) after oral challenge with SE at 2 weeks of age are shown. (B to D) Total secretory IgA antibody titers from mucosal intestinal tracts of the cecum, ileum, and duodenum across four time points postinfection. Antibody measurements were evaluated using an unpaired Mann-Whitney U test between noninfected and SE-infected birds at each time point. Results are presented as geometric means and 95% confidence intervals. *y* axis units are antibody titers on a logarithmic scale. Control serum (*n* = 5) and infected serum (*n* = 13) were collected for each time point.

SE colonization in the infected host was found to be largely confined to cecum sites after oral inoculation, and SE load was not detected in the other internal organs, like the spleen and liver, at all postinfection time points. In the control (noninfected) group, there was no detection of SE in any of the organs (cecum, spleen, and liver) at all four time points measured in the experiment. Colonization levels of SE in the cecum were maintained at approximately 10^7^ CFU/g of cecal content throughout the infection with no sign of bacterial clearance or reduction in bacterial burden, even by 21 days postinfection (dpi) (Fig. 1A). A similar trend in SE colonization persistence in the cecum was also found in a previous infection experiment with 2-week-old chicks using the same genetic lines (data not shown).

### Intestinal sIgA response is insignificant throughout persistent SE infection in the cecal lumen.

**FIG 2:**
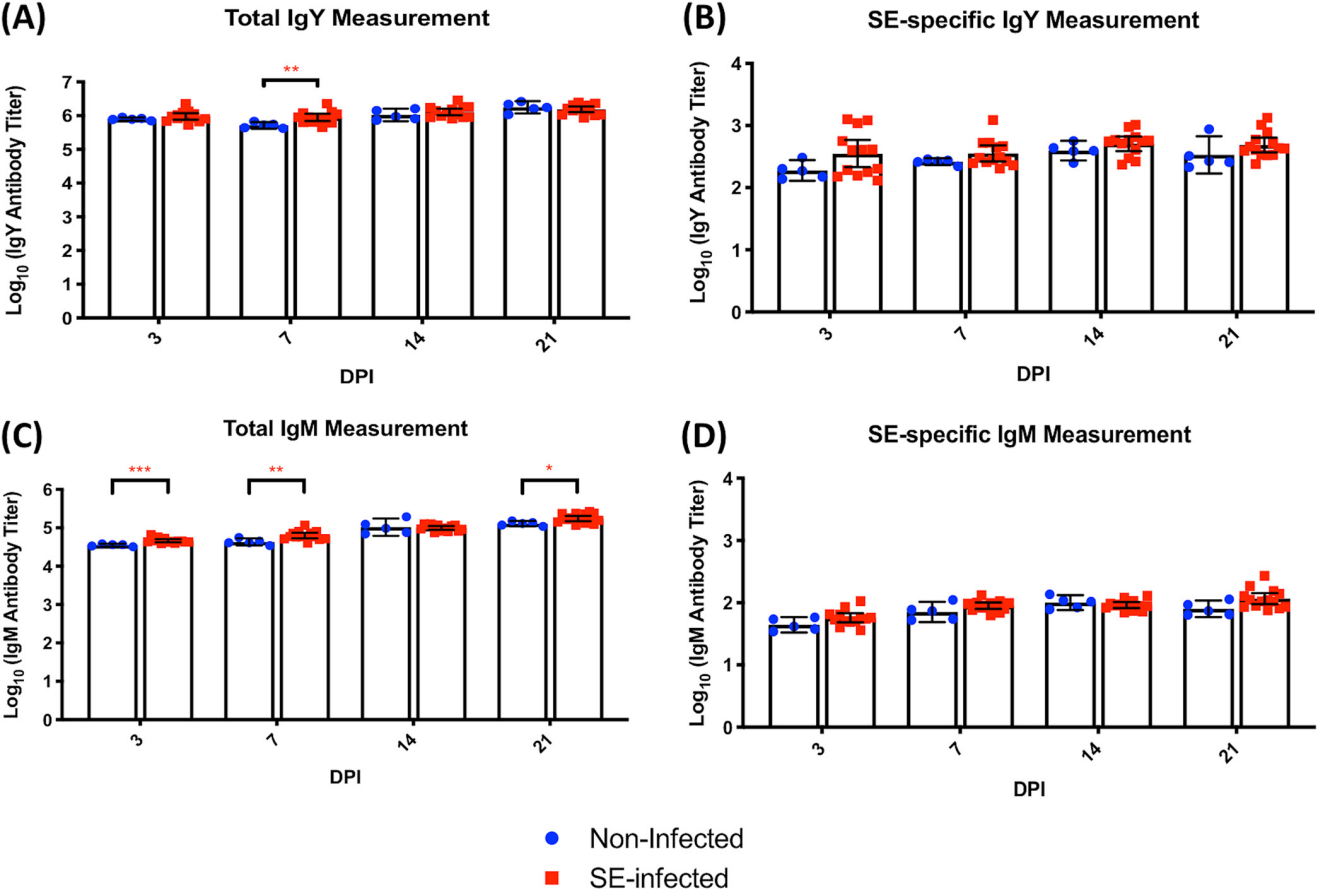
(A and B) Quantification of total circulating IgY and SE-specific circulating IgY, contrasting between SE-infected and noninfected groups. (C and D) Quantification of total circulating IgM and SE-specific circulating IgM in the two groups. Antibody measurements were evaluated using the unpaired Mann-Whitney U test. Results are presented as geometric means and 95% confidence intervals. *y* axis units are antibody titers on a logarithmic scale. Control serum (*n* = 5) and infected serum (*n* = 13) were collected for each time point. *, *P ≤ *0.05; **, *P* ≤ 0.01; ***, *P* ≤ 0.001.

Total secretory IgA (sIgA) titers from mucosal linings of the cecum, ileum, and duodenum were quantified by enzyme-linked immunosorbent assay (ELISA) to assess the role of sIgA response to persistent SE presence in the cecal lumen during the infection. In both the lower and upper sections of the digestive tract, there was no significant elevation in the total sIgA production for the SE-infected group compared to the noninfected group (Fig. 1B to D). To further assess the systemic immune response to SE infection, both total and SE-specific circulatory antibody levels were measured. Positive reactions for SE-specific immunoglobulin isotypes of IgY and IgM were detected in both noninfected and infected chicks. Quantification of SE-specific IgY and IgM, however, showed no significant difference between the two treatment groups across all measured time points (Fig. 2B and D). However, robust total antibody production was detected with a significant increase (*P* < 0.05) in both IgY and IgM antibody levels at different time points of infection in the SE-infected group compared to the noninfected group. At 7 dpi, the total IgY antibody levels in the infected group were significantly higher than the noninfected-host baseline measurement (Fig. 2A). Except for 14 dpi, the total IgM level in the SE-infected chicks also significantly increased at both early (3 and 7 dpi) and late (21 dpi) time points (Fig. 2C).

### Effect of SE colonization persistence on the host cecal tonsil transcriptome.

**FIG 3:**
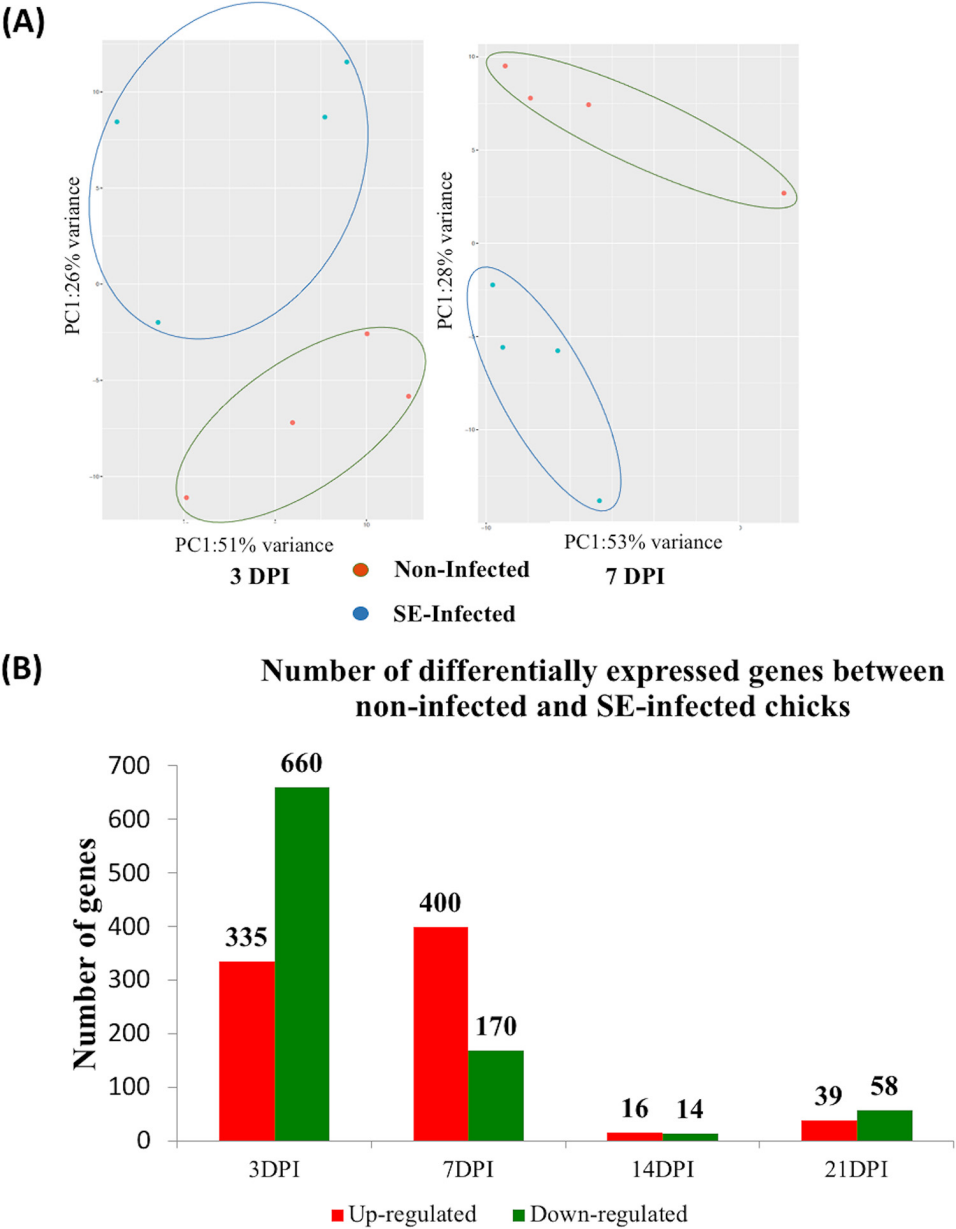
(Continued).

To gain a better understanding of regulation in gene expression at the global level in GALT during host-pathogen interaction that contributed to SE persistence in the gut, the cecal tonsil transcriptome profiles of noninfected and SE-infected birds were compared at each time point. Transcriptome sequencing (RNA-seq) mapping statistics detailing raw read counts, aligned reads, and alignment mapping rates for two treatment groups across four time points are provided in Table 1. Principal-component analysis (PCA) revealed a separate clustering pattern between the two groups at early infection time points of 3 dpi and 7 dpi (Fig. 3A). Differential gene expression analysis was carried out to compare the gene expression profiles between noninfected and SE-infected chicks across the four experimental time points. Large numbers of differentially expressed genes (DEGs) were detected at the early response at 3 dpi and 7 dpi, with totals of 995 and 570 DEGs, respectively. Of those differentially expressed genes, 335 genes were upregulated and 660 genes were downregulated at 3 dpi, while 400 genes were upregulated and 170 genes were downregulated at 7 dpi (Fig. 3B). However, as infection progressed, fewer DEGs were detected between noninfected and SE-infected birds at the lag phase of infection. At 14 dpi, only 30 DEGs were detected (16 upregulated and 14 downregulated) in response to SE infection. A total of 97 DEGs (39 upregulated and 58 downregulated) were detected at 21 dpi. The Venn diagram in Fig. 3C illustrates the overlapped genes that were differentially expressed between noninfected and SE-infected groups across four time points. There were a total of five genes shared across 3, 7, 14, and 21 dpi. Of these five genes, two were annotated and found to be downregulated consistently throughout the four time points in response to SE presence. These two genes encode metallothionein 4 (MT4) and period homolog 3 (PER3), belonging to biological processes that involve response to lipopolysaccharides and interleukin 1 and to circadian regulation of gene expression and negative regulation of transcription from the RNA polymerase II promoter, respectively (Fig. 3D).

**TABLE 1:** Summary statistics of RNA-seq data output

Time point (dpi)	Treatment	No. of reads		Alignment rate (%)
		Raw	Aligned	
3	None	82,276,240	68,992,497	83.85
	SE infection	101,362,688	85,492,936	84.34
7	None	92,925,386	77,636,103	83.55
	SE infection	87,391,716	73,087,219	83.63
14	None	87,586,757	72,826,983	83.15
	SE infection	80,405,982	66,266,548	82.41
21	None	96,620,005	80,561,332	83.38
	SE infection	103,235,105	86,566,725	83.85

### Gene ontology analysis shows enrichment of the bacterium-associated host immune defense response at a late infection stage.

**FIG 4:**
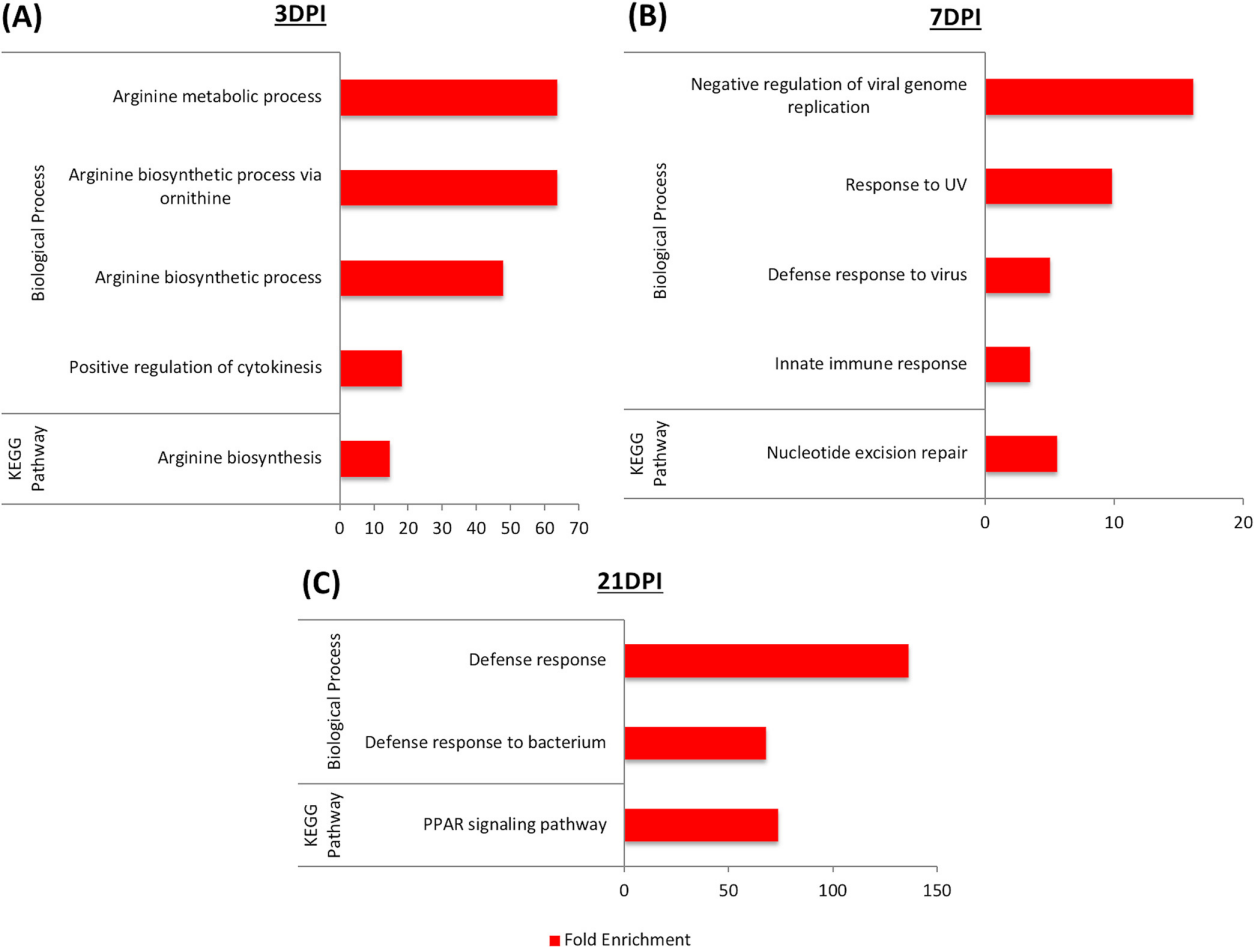
Gene ontology (GO) terms involved in biological processes and KEGG pathways enriched by genes highly expressed in cecal tonsil with SE infection at (A) 3 dpi, (B) 7 dpi, and (C) 21 dpi. There was not any enriched GO term detected at 14 dpi. The DAVID tool was used, with a significance cutoff of >2-fold enrichment and an FDR of <20%.

The DEG list generated was further analyzed for enriched functional terms in both biological processes and KEGG pathways utilizing the Database for Annotation, Visualization, and Integrated Discovery (DAVID) (https://david.ncifcrf.gov/home.jsp). All the upregulated genes associated with SE infection status were first examined for Gene Ontology (GO) analysis. Enrichment of five GO terms was identified (4 biological processes and 1 KEGG pathway) at 3 dpi, with a majority of gene function relating to arginine metabolic synthesis processes along with positive regulation of cytokinesis (Fig. 4A). This result was consistent with our published metabolite data set in which the arginine and proline metabolism pathway was significantly upregulated in the same experimental group of SE-infected chicks (14). At 7 dpi, host response to SE infection becomes more apparent, with enrichment of GO terms that included negative regulation of viral genome replication, defense response to the virus, innate immune response in conjunction with the response to UV, and nucleotide excision repair (Fig. 4B). There were no GO terms significantly enriched in the upregulated genes with SE infection at 14 dpi, possibly due in part to low numbers of differentially expressed genes. Progressive host response to SE infection continued to occur at 21 dpi, with enrichment in general defense responses as well as defense responses specific to the bacterium. Additionally, the peroxisome proliferator-activated receptor (PPAR) signaling pathway was also enriched in response to SE infection (Fig. 4C).

**FIG 5:**
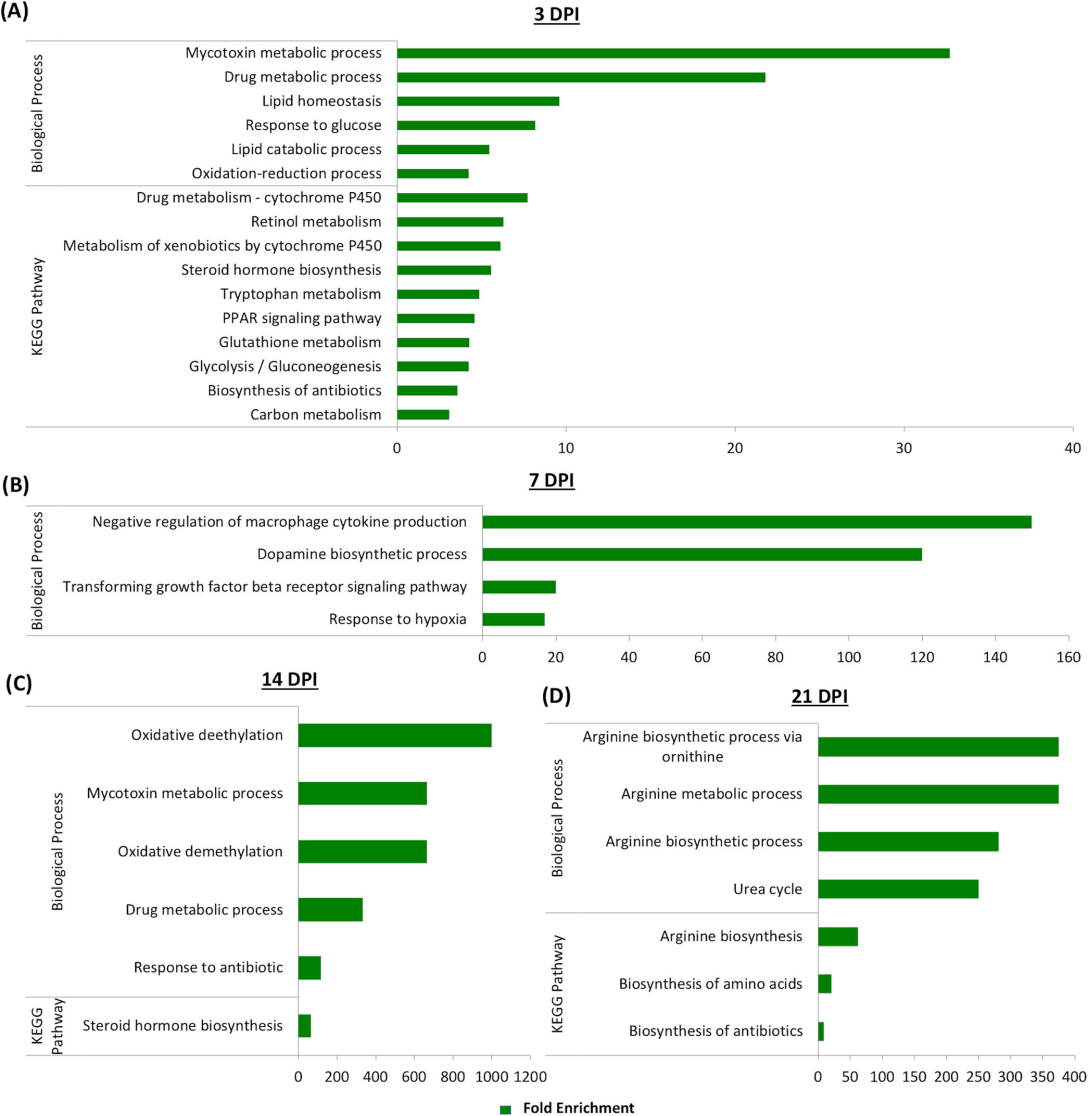
Gene ontology (GO) terms involved in biological processes and KEGG pathways enriched by downregulated genes expressed in cecal tonsil with SE infection at (A) 3 dpi, (B) 7 dpi, (C) 14 dpi, and (D) 21 dpi. The DAVID tool was used, with a significance cutoff of >2-fold enrichment and an FDR of <20%.

Next, GO terms enriched by the downregulated genes in association with SE infection were identified. The greatest number of enriched GO terms were detected at 3 dpi (16 GO terms), including 6 biological processes and 10 KEGG pathways. The majority of enriched GO terms observed were related to host metabolism (mycotoxin metabolic process, drug metabolic process, lipid catabolic process, drug metabolism, retinol metabolism, metabolism of xenobiotic, tryptophan metabolism, glutathione metabolism, and carbon metabolism) (Fig. 5A). Enrichment in a total of 4 biological processes was observed at 7 dpi, with the highest enrichment (150-fold) in negative regulation of macrophages. Enrichment in other biological processes, such as dopamine biosynthesis, transforming growth factor beta receptor signaling, and response to hypoxia, was also detected (Fig. 5B). At 14 dpi, five biological processes (oxidative demethylation, mycotoxin metabolic process, oxidative demethylation, drug metabolic process, and response to antibiotic) and one KEGG pathway (steroid hormone biosynthesis) were enriched (Fig. 5C). Many of the arginine-associated biological processes as well as arginine biosynthesis pathways were enriched by downregulated genes with SE infection status at 21 dpi. Significant enrichment of the urea cycle and the biosynthetic pathway of amino acids and antibiotics was also detected (Fig. 5D).

### IPA highlights host immunological tolerance towards SE infection.

**FIG 6:**
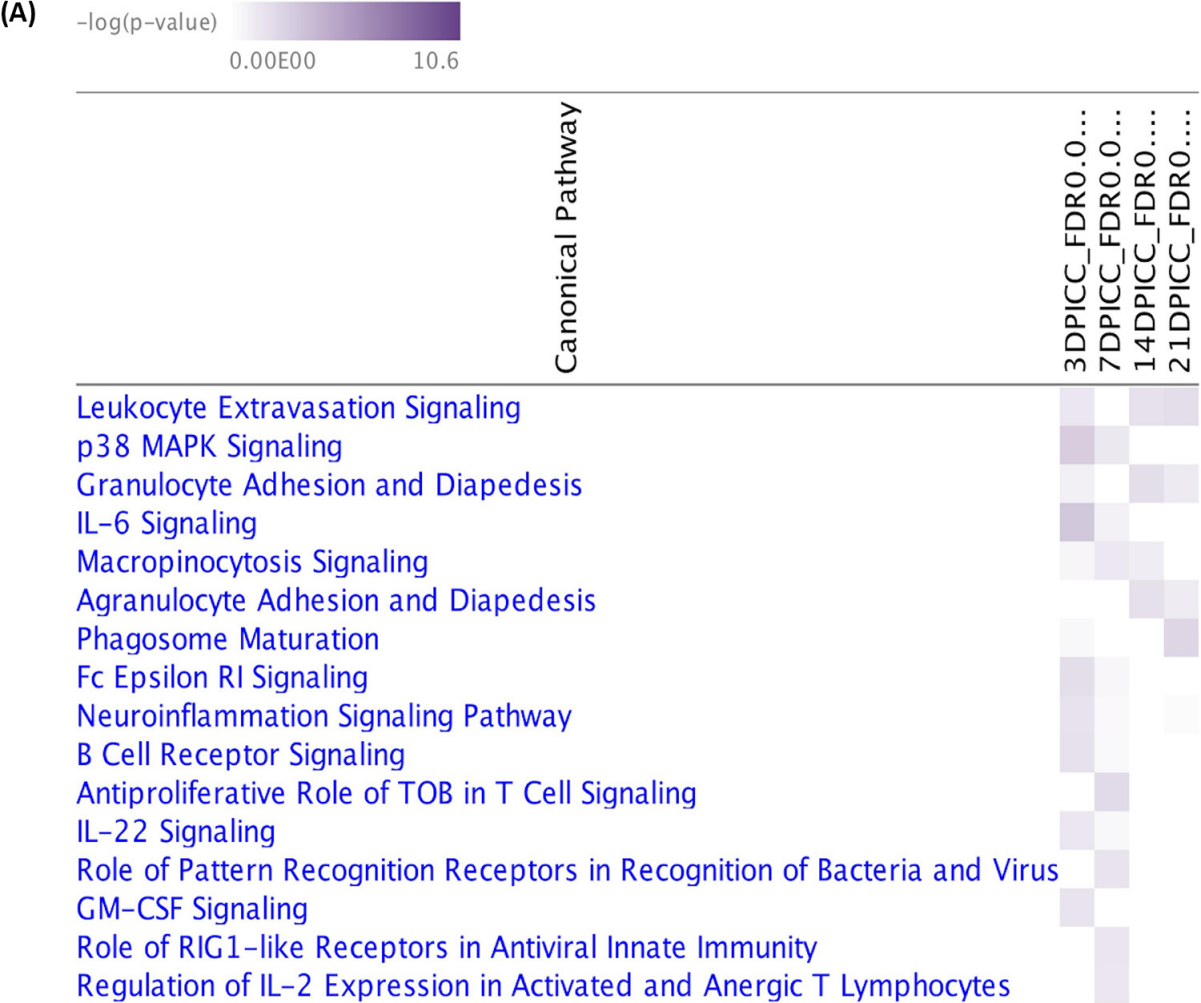
(A) Heat map showing significantly enriched canonical pathways related to immune-associated subcategories predicted by IPA based on gene expression data for SE-infected and noninfected chicks contrasted at four time points (−log *P* > 1.3 or *P* < 0.05). (B to E) Top immune-related pathways predicted to be involved in response to SE infection across four time points. The total number of genes in each pathway is at the top of each bar, with the entire chart comprising downregulated genes (green), upregulated genes (red), and no DEGs in the gene data set input (white). Orange lines represent the –log *P* value of each pathway.

To gain better insight into the host immune regulation in response to SE infection in the gut, pathway analysis targeting immune-related pathways was performed by Ingenuity Pathway Analysis (IPA). Specifically, total represented canonical pathways were filtered to select for cellular immune response and humoral response subcategories in the signal pathways category from the ingenuity canonical pathway. Figure 6A shows a heat map of representative cellular immune pathways from the DEG list, showing the contrast between two treatment groups across four postinfection time points. A total of 16 immune-related pathways were identified as being highly represented at different postinfection time points with pathways relating to subcategories that include four cytokine signaling pathways (interleukin 6 [IL-6], IL-22, granulocyte-macrophage colony-stimulating factor [GM-CSF] signaling, and regulation of IL-2 expression in activated and anergic T lymphocytes), three pathogen-influenced signals (phagosome maturation, the role of pathogen recognition receptors in recognition of bacteria and viruses, and the role of RIG-1 like receptors in antiviral innate immunity), five cellular immune responses leading to host inflammation (macropinocytosis signaling, leukocyte extravasation signaling, granulocyte adhesion and diapedesis, agranulocyte adhesion and diapedesis, and neuroinflammation signaling pathways), two humoral responses (Fcε RI signaling and B cell receptor signaling), one cellular stress and injury response (p38 mitogen-activated protein kinase [MAPK] signaling), and one cellular growth, proliferation, and development response (antiproliferative role of transducer of ERBB2 [TOB] in T cell signaling) (Table 2).

**TABLE 2:** Significantly enriched canonical pathway related to immune-associated subcategory predicted by IPA^*a*^

Canonical pathway (immune related only)	3 dpi		7 dpi		14 dpi		21 dpi	
	*P*	Ratio	*P*	Ratio	*P*	Ratio	*P*	Ratio
Leukocyte extravasation signaling	3.94E−02	12/211	NA	NA	2.05E−02	2/211	1.38E−02	3/211
P38 MAPK signaling	1.64E−03	11/120	4.75E−02	5/120	NA	NA	NA	NA
Granulocyte adhesion and diapedesis	3.06E−05	9/179	NA	NA	1.5E−02	2/179	6.76E−02	2/179
IL-6 signaling	8.42E−04	12/128	1.58E−01	4/128	NA	NA	NA	NA
Macropinocytosis signaling	2.6E−01	4/81	4.39E−02	4/81	8.18E−02	1/81	NA	NA
Agranulocyte adhesion and diapedesis	NA	NA	NA	NA	1.7E−02	2/191	7.57E−02	2/191
Phagosome maturation	3.37E−01	6/148	NA	NA	NA	NA	5.26E−02	3/148
Fcε RI signaling	1.43E−02	9/119	3.09E−01	3/119	NA	NA	NA	NA
Neuroinflammation signaling	2.31E−02	17/311	4E−01	6/311	5.25E−01	1/311	NA	NA
B cell receptor signaling	2.03E−02	12/191	3.81E−01	4/191	NA	NA	NA	NA
Antiproliferative role of TOB in T cell signaling	NA	NA	8.54E−03	3/26	NA	NA	NA	NA
IL-22 signaling	4E−02	3/24	3.27E−01	1/24	NA	NA	NA	NA
Role of pattern recognition receptors in recognition of bacteria and viruses	NA	NA	2.54E−02	6/137	NA	NA	NA	NA
GM-CSF signaling	2.93E−02	6/73	NA	NA	NA	NA	NA	NA
Role of RIG1-like receptors in antiviral innate immunity	NA	NA	3.51E−02	3/44	NA	NA	NA	NA
Regulation of IL-2 expression in activated and anergic T lymphocytes	NA	NA	4.23E−02	4/80	NA	NA	NA	NA

a Significant enrichment was defined as a –log *P* value of >1.3 or a *P* value of <0.05). Ratios are expressed as number of DEGs in a pathway/total number of genes in the pathway. NA, not statistically significant.

The most affected pathways involving early host response to SE infection at 3 dpi were signaling pathways related to IL-6, p38 MAPK, Fcε RI, B cell receptor, neuroinflammation, GM-CSF, leukocyte extravasation, and IL-22 (Fig. 6B). Of six pathways (antiproliferative role of TOB in T cell signaling, the role of pathogen recognition receptors in recognition of bacteria, the role of RIG1-like receptors in antiviral innate immunity, regulation of IL-2 expression in activated and anergic T lymphocytes, macropinocytosis signaling, and p38 MAPK signaling) identified at 7 dpi, upregulation of two pathways, including p38 MAPK signaling (also identified at 3 dpi), was observed (Fig. 6C). Since there were fewer DEGs at the late postinfection time points of 14 dpi and 21 dpi, fewer pathways were enriched, suggesting host adjustment or tolerance to SE presence at these late time points. The involvement of only three pathways (granulocyte, agranulocyte adhesion and diapedesis, and leukocyte extravasation signaling) and two pathways (phagosome maturation and leukocyte extravasation signaling) was observed at 14 dpi and 21 dpi, respectively (Fig. 6D and E).

**FIG 7:**
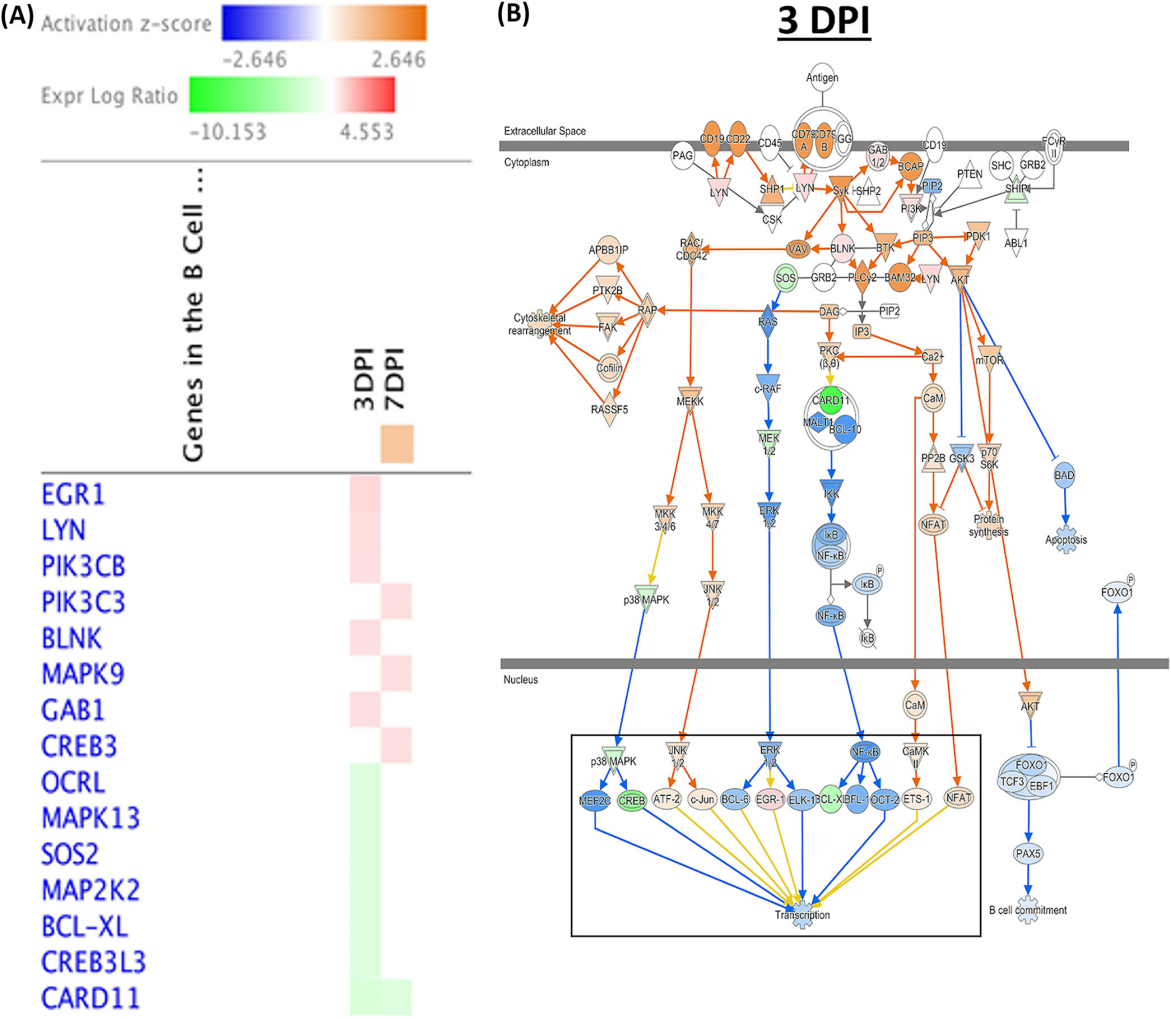
B-cell receptor signaling pathway analysis using Ingenuity Molecule Activity Predictor (MAP). (A) Heat map of genes involved in the B cell receptor signaling pathway generated by contrasting between two treatment groups at early time points (the pathway was not enriched at 14 and 21 dpi). Genes were clustered by similarity on the basis of genes that were upregulated (red), downregulated (green), and not changed (white). This pathway was predicted by IPA to be activated only at 7 dpi (orange). (B) B cell receptor signaling pathway in the SE-infected group at 3 dpi. The highlighted rectangle indicates the downstream inhibition effect of the mapped genes. (C) B cell receptor signaling pathway in the SE-infected group at 7 dpi. The highlighted rectangle indicates the downstream activation effect of the mapped genes. The table shows mapped genes involved in the pathway with respective log_2_ fold changes and FDR values from DEG analysis. Red and green symbols indicate up- and downregulated genes, respectively. Orange and blue nodes indicate genes predicted to be activated and inhibited, respectively.

**FIG 8:**
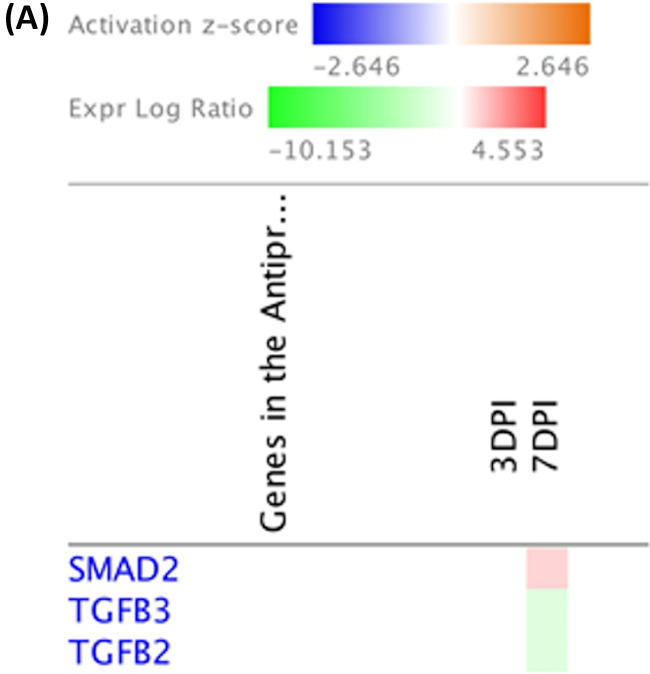
Antiproliferative role of TOB in T cell signaling analysis using Ingenuity Molecule Activity Predictor (MAP). (A) Heat map of genes involved in the antiproliferative role of TOB in the T cell signaling pathway generated by contrasting SE infected and noninfected groups at early time points (the pathway was not enriched at 14 and 21 dpi). Genes were clustered by similarity on the basis of genes that were upregulated (red), downregulated (green), and not changed (white). (B) Involvement of the antiproliferative role of TOB in the T cell signaling pathway in the SE-infected group at 7 dpi. The table shows mapped genes involved in the pathway with respective log_2_ fold change and FDR values from DEG analysis. Red and green symbols indicate up- and downregulated genes, respectively. Orange and blue nodes indicate genes predicted to be activated and inhibited, respectively.

**FIG 9:**
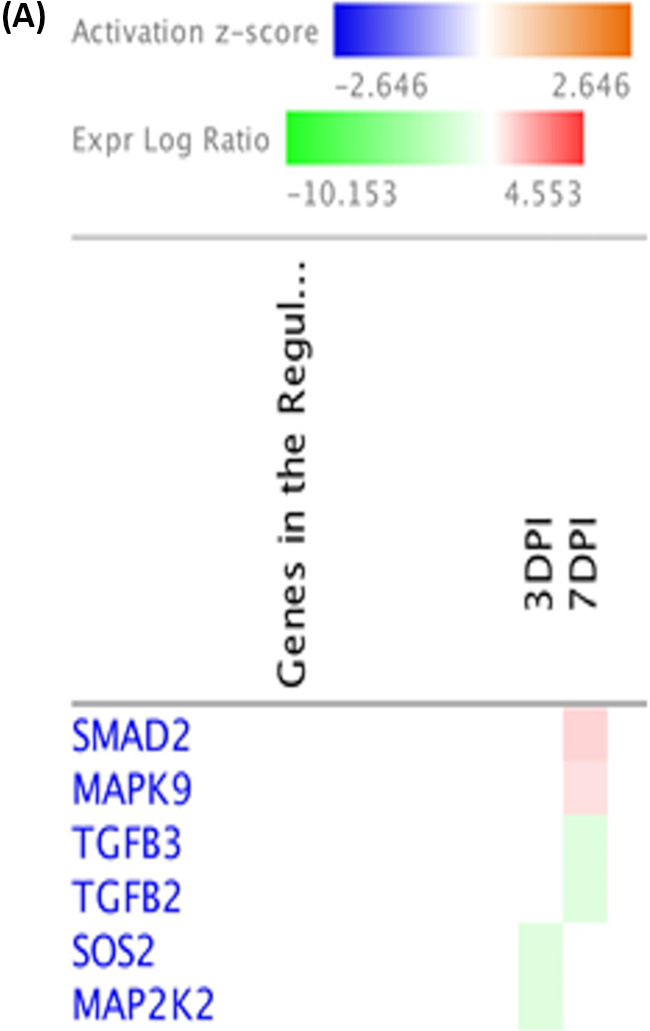
Regulation of IL-2 expression in activated and anergic T lymphocytes using Ingenuity Molecule Activity Predictor (MAP). (A) Heat map of genes involved in the regulation of IL-2 expression in activated and anergic T lymphocytes generated by contrasting SE-infected and noninfected groups at early time points (the pathway was not enriched at 14 and 21 dpi). Genes were clustered by similarity on the basis of genes that were upregulated (red), downregulated (green), and not changed (white). (B) Regulation of IL-2 expression in activated and anergic T lymphocytes in the SE-infected group at 7 dpi. The tables show mapped genes involved in both activated T cells and anergic T cells with their respective log_2_ fold change and FDR values from DEG analysis. Red and green symbols indicate up- and downregulated genes, respectively. Orange and blue nodes indicate genes predicted to be activated and inhibited, respectively.

Through the utilization of molecule activity predictor (MAP) tools in IPA, predictions of activation or inhibition of immune functional pathways were generated based on differential gene expression levels from two treatment groups. MAP predicts the upstream and downstream effects of the mapped genes on the candidate pathway and predicts the overall state of the pathway. B cell receptor signaling was predicted to be active at 7 dpi following host response to SE infection (Fig. 7A). Changes to upstream genes dictating its activation were upregulation of PIK3C3, MAPK9, and CREB3 as well as downregulation of CARD10 based on gene expression levels between two treatment groups (Fig. 7B). Although at 3 dpi, significant numbers of DEGs (12 DEGs of 191 total genes) were found to participate in the B cell receptor signaling pathways, transcription leading to B cell activation was inhibited based on MAP prediction. The MAP tool also predicted the activation of the antiproliferative role of TOB in T cell signaling pathways at 7 dpi (Fig. 8A), which results in an unresponsive state of T cells in SE infection. The main change in an upstream gene contributing to this pathway was upregulation of expression of SMAD2 (log_2_ fold change = 0.938) (Fig. 8B and 9). Regulation of IL-2 expression was predicted to be active in activated T cells as well as inhibited in anergic T cells. Based on pathway prediction at 7 dpi, there was slight upregulation in MAPK9/JNK gene expression (log_2_ fold change = 0.411), which resulted in active transcription of the IL-2 gene in the activated-T-cell population (Fig. 9). In parallel, inhibition of IL-2 expression in anergic T cells was also predicted due to the TOB/SMAD signaling cascade contributed by SMAD2 transcript level (log_2_ fold change = 0.938).

## DISCUSSION

The development of the gastrointestinal tract and its associated immunological functions in an avian host is influenced by genetic, nutritional intake as well as exposure to other exogenous factors after hatching (15). The first 2 weeks after hatching is a critical time for the development of the gut-associated immune system and gut microbiota in chicks. Therefore, the 2-week infection model provides a unique opportunity to assess the functional role of the host defense program, specifically in gut-associated organs, against intestinal pathogens. The cecal tonsil is the main secondary lymphoid tissue located at the proximal end of the cecum and a component of the GALT, and it plays an important role as an immunological monitor of the luminal content in the gut (16). In the present study, we explored the underlying colonization persistent mechanism of SE infection by assessing the immunological functions and regulation of gene expression level in cecal tonsils using RNA-seq technology in a model of infection in 2-week-old chicks. Dynamic changes that occurred in a major lymphoid organ within the digestive tract of the chicken host at the transcriptome level offered novel insights into host-pathogen interaction occurring at the primary site of SE colonization. Correlation between the transcriptome signature of humoral immunity and the titers of antibody produced at both mucosal and systemic sites was further evaluated in the current study.

Cecal tonsil transcriptome analysis revealed a significant number of DEGs early in the host response (3 and 7 dpi); however, as infection progressed, a reduced number of DEGs were observed between infected and noninfected birds. These data suggested either host recovery from infection or adjustment to the presence of SE. The persistent presence of colonizing SE in cecum at late postinfection time points (14 and 21 dpi) suggested that the latter is the more likely scenario. Among the differentially expressed gene (DEGS), metallothionein 4 (MT4) was found to be consistently downregulated during SE infection at all four postinfection time points. MT family genes encode low-molecular-weight, cysteine-rich metal-binding proteins that play an important physiological role in the maintenance of metal homeostasis, particularly zinc regulation, in all organisms (17). MT expression levels are induced by numerous stimuli, including stress conditions, oxidative stress, heavy metals, and toxins. Differential regulation of their gene expression during bacterial and virus infection has been implicated to affect host immune response (18–20). Specifically, MT gene expression is linked to the host inflammation response mediated by bacterial lipopolysaccharide (LPS). Leghorn birds injected with either LPS or IL-1 exhibit increased liver metallothionein concentrations during the acute-phase immune response (21, 22). In the current model, however, the MT4 gene was observed to be downregulated at all four time points despite persistent SE presence. An alternative explanation for the observed outcome could be linked to zinc regulation, as synthesis by MT genes, which encode a metal-binding protein family, has a direct impact on zinc homeostasis in the host. The relevance of zinc in host-pathogen interaction had been described by Ammendola et al. (23) and Wu et al. (24). While zinc acquisition by Salmonella was found to be important for its intracellular survival in mice (23), Salmonella infection in MT gene knockout cells displayed increased zinc accumulation as well as pathogen colonization (24). Hence, we speculated that downregulation of the MT4 gene in the host might be part of the SE pathogenesis strategy to potentially increase zinc resources and thus enhance its survival while further establishing colonization in the chicken gut.

Our results in the current chicken model suggested that persistent Salmonella colonization in the gut might be driven by the host tolerogenic adaptive immune response with predicted canonical pathway enrichment of TOB in T cell signaling. T cell activation requires efficient ligation of the T cell receptor (TCR) by antigen to initiate the process of immune cell clonal expansion specific for the invading pathogen (25). However, the T cell activation process in the host is under tight regulation to prevent an excessive immune response and reactivity against self-antigens. TOB is a member of an antiproliferative gene family (26) and plays an important role in the negative regulation of T cell proliferation. TOB is constitutively expressed in resting T cells and downregulated after antigen-specific stimulation, subsequently lowering the threshold for T cell activation. Downregulation of TOB is mandatory for T cell activation and expansion (27). Enrichment in the antiproliferative function of the TOB pathway at 7 dpi during SE infection indicated the inhibition of T cell cycle progression, further supporting potential anergic T cell response.

T cell anergy is part of the host tolerance mechanism by which antigen stimulation fails to activate T cell clonal expansion and remains functionally unresponsive (28). T cell anergy could be a result of either insufficient antigen activation signal or the presence of inhibitory signals. Based on pathway analysis, SE-associated antigen upregulated the JNK transcript level at 7 dpi, consequently leading to predicted IL-2 gene expression in the activated T cells. Although regulation of IL-2 expression in activated T cells suggested partial T cell activity, the expression of the TOB/SMAD signaling cascade in anergic T cells was also predicted in parallel, which accounted for antigen-specific unresponsiveness and blockage of IL-2 transcription. In the anergic T cells, upregulation of SMAD2 enhances the interaction of the SMAD complex with TOB, which effectively inhibits IL-2 expression, thereby leading to the negative regulation of the T cell immune response. Hence, there appears to be a tug-of-war for regulation of IL-2 expression between effector T cells and regulatory T cells (29).

IL-2 is a major cytokine involved in the growth, proliferation, and differentiation processes of immune cells, particularly antigen-activated T cells, and is also known as the T cell growth factor (30). Successful pathogen clearance from the host requires substantial production of IL-2 and efficient T cell proliferation. A weak SE-associated antigen signal for activating the effector T cells in the current model could be a plausible explanation for the predicted tolerogenic immune response where regulatory T cells win the tug-of-war for regulating IL-2 expression. Maximal induction of IL-2 expression requires the involvement of the multiple pathways, including ERK (extracellular signal regulated kinase), JNK (c-Jun N-terminal kinase), NF-κB, and NFAT (nuclear factor of activated T cells) (31). In the current model, only the JNK transcript was slightly upregulated at 7 dpi, indicating insufficient antigen signal to trigger the transcript level of other pathways for maximal activation of IL-2 expression.

Active involvement of regulatory T cell (T_reg_) regulation in suppressing the T cell response during persistent SE infection in broiler chickens was demonstrated in a study by Shanmugasundaram et al., in which an increased T_reg_ cell population was detected after SE infection in cecal tonsil of the host (32). Additionally, there is a potential linkage between downregulation of MT4 gene expression and IL-2 transcription giving rise to the T cell tolerance response. The immune-modulatory role of the MT gene has been implicated in the development and functionality of immune cells during infection (20). Induction of MT expression was demonstrated to be essential for the production of IL-2. Lack of MT expression in MT-null mice leads to substantially lower production of IL-2 as well as reduced T cell response upon stimulation with mitogen (33). Therefore, we further speculate that consistent downregulation of the MT4 gene during SE infection in chicks may have a consequential effect on host defense response, with a potential failure to induce effective IL-2 expression.

Secretory IgA is a major class of antibodies found in the intestinal mucosal secretions and functions primarily to prevent microbial pathogens from achieving access or attachment to the intestinal epithelium or breaching the intestinal barrier (34). Intestinal sIgA, therefore, plays a protective role against SE colonization and invasion of the intestinal mucosal surfaces. Here, the inability of the host to clear or reduce SE colonization from the gut was supported by insignificant production of gut secretory IgA antibodies and highlighted the inadequate mucosal immunity protection. Since extraintestinal dissemination of SE was not observed (spleen and liver), the role of host systemic antibody response was also investigated. An effective systemic antibody response can limit or prevent the translocation of the intestinal pathogen to systemic sites (35). An elevation in titer of total circulatory antibody for both IgY (7 dpi) and IgM (3,7 and 21 dpi) in SE-infected birds suggested that the cross talk between SE and the chick host elicits a systemic humoral immune response. However, the kinetics of SE-specific antibody production in infected birds was insignificant compared to those in the noninfected birds, revealing minimal induction of the pathogen-specific circulatory antibody response. This could be contributed to by the limitation of the SE colonization site at the intestine in the current model.

The presence of SE-specific antibody in nonchallenged birds also revealed the involvement of passive immunity. Maternal antibodies are passively transferred from the hen through the egg yolk and eventually are absorbed into the circulatory system of the newly hatched chicks (36). Maternal antibodies confer partial protection against specific pathogen infection that the dam has been exposed to, through transfer of antigen-specific protection to new hatchlings. The presence of maternal antibodies within the circulatory system of the chick host can persist until sometime between 2 and 4 weeks of age, when it gets catabolized from the system (37–40). Preexisting Salmonella-specific maternal antibodies in the circulation of the chicks could prevent SE from disseminating systemically in the host. In agreement with antibody titer measurement, cecal tonsil host gene expression analysis demonstrated a failure in the host to initiate a substantial amount of humoral immune response during the initial phase of SE colonization in the gut with nonactive transcription of B cell receptor (BCR) signaling pathway at 3 dpi. Instead, the active transcription level of BCR signaling was initiated at 7 dpi, which coincides with a spike in total circulatory antibody production (both IgY and IgM) in the infected birds. BCR signaling is a key initiating step that begins the process of development, proliferation, survival, and activation of the B cell (41). Upon stimulation with SE, activation of the BCR signaling cascade could initiate the cell cycle progression and proliferation of B cells specific for recognition of SE, leading to differentiation of plasma B cells as well as memory B cells. However, the sustainability of active BCR signaling to continue with the process of generating the population of antibody-producing cells or effector B cells was not observed in the lag phase of infection despite persistent SE presence in the gut.

The host gene regulatory networks revealed by dynamic cecal tonsil transcriptomic analysis in our study supported the predicted immunological tolerance state of SE infection in an infection model in 2-week-old chicks. Enriched GO terms related to the innate immune defense program and activation of multiple cytokine signaling pathways indicated a host inflammatory response at the initial phase of infection. In addition, upregulation of leukocyte extravasation signaling during the early response also marked the infiltration of the leukocyte towards the site of infection at the cecal mucosa.

Although the early onset of innate immune response was sufficient to control the pathogen invasion process and limit the spread of the pathogen beyond the intestinal barrier, SE persisted in the gut throughout infection. Initiation of the innate immune signaling cascade that occurred during the cross talk between host and pathogen also subsequently reshaped the activation and modulation of the adaptive immunity. Our results suggest that consistent downregulation of the MT4 gene during SE infection highlighted potential pathogen manipulation in host gene regulation to enhance its survival through the utilization of host zinc resources and the impairment of the cellular mechanism of defense. Despite orchestration of early host defense response to infection, the persistent high bacterial load in cecum suggests that once SE colonization is established in the gut, activation of the innate immune response alone is not sufficient to eradicate the pathogen.

Furthermore, participation of the potential tolerogenic adaptive immune response in SE presence was evident in the current study. Enrichment of the TOB/SMAD2 complex signaling pathway and the tug-of-war for regulation of IL-2, which is predicted to be won by the regulatory T cells over the effector T cells, were factors contributing to immunological tolerance. Since there is a direct interlinked signaling pathway between B and T cells, a delay in early activation as well as the inability to sustain active BCR signaling after 7 dpi could subsequently weaken the B cell signaling and antigen uptake that are essential for downstream T cell activation. Insignificant production of intestinal secretory IgA throughout the upper and lower sections of the intestinal tract further enhanced the establishment of SE presence in the gut. Collectively, our data suggest that there might be a failure in the host to induce an appropriate immune response while the host regulatory mechanism appears to be redirected towards a tolerance state that enables SE persistence in the gut.

## MATERIALS AND METHODS

### Animal experiments.

Newly hatched layer chicks from the highly inbred genetic line UCD003 were received from the University of California, Davis, Hopkins Avian Facility. After cloacal swabbing of all control chicks to ensure that they were Salmonella free, the chicks were transferred and separated into two housing chambers with identical environmental conditions. All chicks were housed on the concrete floor with pine shavings in a temperature-controlled setting throughout the study with *ad libitum* access to water and nonmedicated commercial feed. At 2 weeks of age, one group of chicks was orally inoculated with 1 × 10^9^ CFU of a kanamycin- and carbenicillin-resistant strain of S. Enteritidis (kindly provided by Andreas Baumler), while another group received phosphate-buffered saline (PBS) as the control. The amount of the bacterial inoculum was confirmed by serial dilution plating.

Following infection, chicks from both control and SE-infected groups were euthanized via carbon dioxide asphyxiation at 3, 7, 14, and 21 dpi to harvest organs, and each organ collected was weighed before homogenization. SE in chicks was enumerated by plating serial 10-fold dilutions of the homogenized cecal contents, spleens, and livers on MacConkey agar plates that contain the selective antibiotics kanamycin and carbenicillin. The geometric mean of the SE bacterial load recovered from infected chicks at each of the postinfection periods was calculated as CFU per gram of the organ. Cecal tonsil was collected in RNAlater solution at all measured postinfection time points for RNA-seq analysis.

All animal experiments were approved by the Institutional Animal Care and Use Committees at the University of California, Davis (IACUC no. 19272). All experiments performed were in accordance with IACUC guidelines and regulations.

### Detection of intestinal secretory IgA and circulatory antibody response with ELISA.

To quantify the total secretory IgA titer, the intestinal lumen was exposed, and the mucus layer was collected by scraping off the mucosal surface of the intestinal sections (cecum, ileum, and duodenum). For serum sample collection, blood was collected from the leg vein of the bird postmortem and incubated at room temperature for approximately 1 h to allow the blood to clot. Both the mucus samples and clotted blood samples were centrifuged at 5,000 × *g* for 10 min. The supernatant was collected and stored at −20°C for ELISA. Total antibody levels were determined by first coating the 96-well plate with 1 μg/ml of affinity-purified goat anti-chicken IgY, IgM, or IgA antibody in coating buffer for 1.5 h.

ELISA was then performed following the protocol of the Bethyl Laboratories chicken ELISA quantitation kit (Bethyl Laboratories, Inc., Montgomery, TX). Briefly, the plate was washed five times with wash buffer between steps and blocked with blocking buffer for 30 min after the first wash. All incubation steps were carried out at room temperature unless stated otherwise. Diluted replicate mucosal and serum samples and a known concentration of chicken reference serum as standards were incubated in each well for 1 h. Horseradish peroxidase (HRP)-conjugated goat anti-chicken IgY, IgM, or IgA diluted to 1:12,000 was added to all wells and incubated for 1 h. Tetramethylbenzidine (TMB) substrate was then added to all wells, and wells were incubated for 15 min before the reaction was stopped with stop solution as the last step of the protocol.

The optical density at 450 nm (OD_450_) was read by the microplate reader. For the determination of the SE-specific-antibody level, each well of the 96-well plates except for the standard wells was precoated and incubated overnight at 4°C with coating buffer containing 2.5 μg/100 μl of SE LPS antigen (Sigma-Aldrich; L6011). For the standard curves, wells were coated with 1 μg/ml of affinity-purified goat anti-chicken IgY or IgM antibody in the coating buffer and incubated for 1.5 h. The rest of the protocol followed the same procedure as the total-antibody ELISA. The Mann-Whitney test was used to compare the antibody levels between the two treatment groups (*P* < 0.05) for both total antibody and antigen-specific antibody.

### RNA isolation and library construction.

Total RNA from cecal tonsils organ was isolated using TRIzol (Thermo Fisher catalog no. 15596026) with the standard phenol-chloroform method and a precipitation step with 100% ethanol (EtOH). The RNA pellet was dissolved in nuclease-free water and treated with DNase I (Thermo Fisher catalog no. EN0521). RNA library preparation was performed according to the instruction manual for the NEBNext Ultra Directional RNA library preparation kit for Illumina (New England Biolabs [NEB] catalog no. E7420S). RNA-seq libraries were then validated and quantified with an Agilent Bioanalyzer high-sensitivity kit (Agilent catalog no. 5067-4626) and a Qubit dsDNA HS assay kit (Thermo Fisher catalog no. Q32854). Samples were submitted to the UC Davis Genome Center, DNA Technology Core Facility, for 100-bp paired-end sequencing on the Illumina Hiseq4000 platform with a minimum sequencing depth of 30 million reads per sample.

### RNA-seq data analysis.

Data analyzed from RNA-seq libraries consisted of 4 randomly selected samples from 2 treatment groups at each of 4 postinfection time points (total number of samples = 32). Raw reads were trimmed using Trim Galore version 0.4.1 with the “—paired” setting and then aligned with the chicken reference genome Galgal5 using Tophat version 2.1.1. The Ensembl annotation was provided during alignment using the default setting. Alignments were filtered using SAMtools to remove those with a mapping quality (MAPQ) value below 30. Gene expression was quantified using htseq-count 0.6.1. Finally, differentially expressed genes were determined using DESeq2, at a combined cutoff of a false discovery rate (FDR) of <0.05 and a fold change of >1.2. Overrepresentation of functional categories was performed with DAVID (NIAID, NIH) at a significant cutoff of an enrichment of >2-fold and an FDR of <20%. DEGs from contrasts between two treatment groups across four time points were used for pathway analysis with Qiagen’s Ingenuity Pathway Analysis software (IPA; Qiagen).

### Data availability.

Sequence data are available under the accession number PRJNA663303 in the Sequence Read Archive (https://www.ncbi.nlm.nih.gov/sra/).
